# Disparities in cervical cancer screening participation in Iran: a cross-sectional analysis of the 2016 nationwide STEPS survey

**DOI:** 10.1186/s12889-020-09705-2

**Published:** 2020-10-22

**Authors:** Rozhin Amin, Ali-Asghar Kolahi, Nader Jahanmehr, Ali-Reza Abadi, Mohammad-Reza Sohrabi

**Affiliations:** 1 Department of Community Medicine, School of Medicine, Shahid Beheshti University of Medical Sciences, Tehran, Iran; 2 Social Determinants of Health Research Center, Shahid Beheshti University of Medical Sciences, Tehran, Iran; 3 Prevention of Cardiovascular Disease Research Center, School of Management and Medical Education, Shahid Beheshti University of Medical Sciences, Tehran, Iran

**Keywords:** Cervical cancer screening, Disparities in health, Equity in health, Health service utilization, Iran, Pap smear test

## Abstract

**Background:**

One of the most important concerns in every healthcare system is the elimination of disparities in health service utilization and achievement of health equity. This study aimed to investigate the disparities in cervical cancer screening participation in Iran.

**Methods:**

A cross-sectional study was conducted using data from the National Non-Communicable Risk Factors Survey in 2016 (STEPs 2016). Data on cervical cancer screening in addition to demographic and socio-economic factors from 15,975 women aged 18 and above were analyzed. The distribution of surveyed women with regard to cervical cancer screening practice was described. Chi square and logistic regression were used to assess the association of demographic and socio-economic factors with cervical cancer screening participation.

**Results:**

Overall, 52.1% of women aged 30–59 years, had undergone cervical cancer screening at least once in their lifetime. Participation rate in cervical cancer screening programs varied between provinces; ranging from 7.6% in Sistan and Baluchestan to 61.2% in Isfahan. Single marital status, illiteracy, being employed, and having no insurance coverage were associated with lower participation. Age and area of residence were insignificant predictors for participating in cervical cancer screening program. Analysis of the cervical cancer uptake rates across the socio-economic levels revealed that the service is less utilised by high income groups.

**Conclusions:**

Participation in cervical cancer screening program in Iran is not optimal and could be improved. With regard to the distribution of cervical cancer screening practice, social and geographical disparities indicate the need for further research and more comprehensive strategies in order to reduce them.

## Background

Cervical cancer was the fourth most common cancer and the fourth leading cause of cancer deaths in women worldwide in 2018; accounting for 570,000 new cases and 311,000 deaths, equal to 6.6 and 7.5% of the global cancer burden respectively [1]. Rates are significantly higher in developing countries. Over 80 % of new cases and cervical cancer deaths occur in less developed regions of the world [2, 3].

Iran is among the countries with low incidence rate for cervical cancer. The estimated average age-standardized incidence rate for the country is 2.5 per 100,000 women. Despite the low rate, mortality to incidence ratio is relatively high at about 42%. The advanced clinical stage, at which most cervical cancers are identified, is responsible for the poor prognosis and higher mortality rates [4, 5].

The standard secondary prevention method for cervical cancer is cervical cytology, which has been established for more than four decades in most countries [6]. However, studies have documented large discrepancies in the effect of cytology screening programs on cervical cancer rates reduction [7, 8]. The extend of participation of at risk women in screening program has been considered as the primary contributor to these differences [9]. In Iran, the nation-wide cervical cancer screening program has been in operation since 1980s. The program is performed on an opportunistic basis, offering annual cytology smears to women in the targeted age range, 3 years after the onset of their sexual activity. The screening will be continued at 3 years’ intervals after three consecutive normal results. The service is provided by midwives through primary public health care settings, and by gynaecologists in private clinics. The recommended target population was primarily defined as women aged 20 to 65, but was later revised to the age range of 30 to 59 in 2017. However, tests outside the program is common specially in private settings. Basic health insurance plans cover most direct costs and patient’s contribution is low [10, 11].

There are different barriers deterring women from participating in cervical cancer screening program including demographic, structural, personal, and socio-cultural factors. These factors will eventually result in differences in cervical cancer screening uptake which tend to be seen as inequities in the service use [12, 13]. Existing literature has highlighted factors like age, marital status, embarrassment, lack of knowledge, and socio-cultural beliefs around cervical cancer as the main causes of non-attendance in cervical cancer screening program in Iran [14, 15]. However, despite the global growing concern about health equity in recent decades, there are limited studies examining regional and socio-economic variations in cervical cancer screening uptake in the country. Hence, this study sought to assess the disparities in cervical cancer screening participation in Iran according to the data obtained from the National Survey of the Risk Factors of Non-Communicable Diseases (STEPs 2016), and to identify socio-demographic factors that predict the participation in cervical cancer screening program.

## Methods

A study was performed using data from the National Survey of the Risk Factors of Non-Communicable Diseases in Iran (STEPs 2016). This cross-sectional household survey was conducted at the national level through personal interviews at homes. To estimate the sample size, a proportional to size sampling plan was adopted using the least populated province of the country as the basis and calculating the sample size of others according to the population ratio of each province to the referenced one. Weighting methods were used for provinces with sample size of 800 or more. In order to control for sampling effect and non-response error, each provincial sample size was increased by 10%. Ultimately 3105 clusters, each consisting of 10 households, were selected from urban and rural areas by designing a systematic cluster random sampling frame for all provinces. Additional details on the methodology of the survey is available elsewhere [16].

Totally 31,050 participants aged 18 or older were interviewed nationwide. Among the interviewees 15,975 participants were women. All women were asked the following pap smear screening question: “Have you ever had a Pap test for cervical cancer screening?”

### Variables

In order to assess the determinants of participation in cervical cancer screening program, the outcome variable was defined as having ever undergone cervical cancer screening (yes/no). The independent variables selected for the analysis included age, marital status, education level, employment status, residing area, health insurance coverage, and socio-economic status. For easier interpretation, all variables were categorized. Age was categorized in 10-year age groups, and education level was classified by number of years attained. Marital status was divided into single, married, divorced, and widowed. Additionally, employment status was defined as either employed, unemployed, retired, or student. Residing area was categorized as urban or rural area, and insurance coverage as having been covered or not.

In order to measure socio-economic inequality in cervical cancer screening participation, a single living standard variable was produced through principal component analysis (PCA) statistical method and by using data from household assets. Asset variables included home, car, kitchen, bathroom, phone, TV, air cooler, central heating system, refrigerator, freezer, oven, vacuum cleaner, washing machine, dishwasher, personal computer, mobile phone, internet access, and having access to water and gas pipelines. To check if the data is suitable for data reduction, the following two tests, Kaiser-Mayer-Olkin (KMO) Measure of Sampling Adequacy and Bartlett’s test of sphericity, were carried out prior to PCA. The value of KMO obtained was 0.787 and the result of Bartlett’s test of sphericity was significant, therefore PCA was appropriate [17]. In the analysis performed, seven components had eigenvalues greater than 1 and explained 64% of the variation in the data. A socio-economic status variable was created by retaining the principal component with the largest eigenvalue. The constructed variable was then divided into five quintiles, where the first quintile and the last quintile represented the poorest and the richest socio-economic levels respectively.

### Statistical analysis

Descriptive statistics (absolute and relative frequencies) were used to describe the distribution of women aged 18 and over with regard to cervical cancer screening practice. To examine the association between socio-demographic characteristics and the cervical cancer screening adherence, the Chi-Squared test was performed. Logistic regression analysis was undertaken for calculating adjusted socio-demographic disparities. To this purpose, the analytic population was restricted to women aged 30–59 years, for whom cervical cancer screening is recommended in the latest edition of the national guideline. The estimates were analysed by using IBM SPSS Statistics for Mac, version 26 (IBM Corp., Armonk, N.Y., USA), assuming a significance level of α < 0.05.

Since the data was collected by trained in-home interviewers based on a standardized protocol, the proportions of missing values were low. The missing rates ranged between 1 to 5% across all variables in the dataset, therefore the impacts of the missing values on the quality of statistical inferences was regarded as insignificant [18].

## Results

Data from 15,975 participants were analyzed. The characteristics of the study participants are shown in Table 1. Most women in our sample were aged 30–39, married, and had elementary education (1–6 years). The majority were residing in urban areas and unemployed. Over 90 % had basic primary health insurance, yet less than a third were covered under the complementary insurance plan.

**Table 1:** Descriptive Statistics of Women aged 18 and above by Cervical Cancer Screening Participation

Sociodemographic variable	n	%	Participation rate %	***p***-value
**Age group**				0.05
18–29	3294	21.6	24.9	
30–39	3696	24.2	51.1	
40–49	3016	19.8	55.9	
50–59	2488	16.3	49.1	
60–69	1672	11.0	32.9	
+ 70	1094	7.2	16.6	
**Marital status**				< 0.001
Single	1967	12.6	1.9	
Married	11,554	73.8	50.7	
Widowed	1730	11.0	25.4	
Divorced	412	2.6	42.6	
**Education**				< 0.001
Illiterate	3305	21.5	24.4	
1–6 years	5852	38.0	47.4	
6–12 years	3858	25.0	49.1	
> 12 years	2387	15.5	39.7	
**Employment**				< 0.001
Unemployed	13,262	84.6	42.9	
Employed	1467	9.4	42.7	
Retired	326	2.1	55.4	
Student	622	4.0	5.7	
**Primary Insurance**				< 0.001
No	1035	6.6	32.1	
Yes	14,682	93.4	42.3	
**Complementary Insurance**				< 0.001
No	12,270	78.7	39.0	
Yes	3312	21.3	51.0	
**Residence**				< 0.001
Urban	11,203	70.1	44.7	
Rural	4772	29.9	34.3	
**Socio-economic Quintiles**				< 0.001
Lowest	3195	20.0	53.1	
Low	3195	20.0	47.1	
Middle	3198	20.0	41.6	
High	3194	19.9	32.2	
Highest	3193	19.9	24.8	
**Total**	15,975		41.6	

*N* number of women in each category, *%* percentage of women in each category; Participation rate, percentage of women in each category who reported undergoing cervical cancer screening; *P*-value, obtained from cross-tabulation between each category and cervical cancer screening

Overall, 41.6% of women aged 18 and over reported ever had a cervical cancer screening. The highest proportion was found in women aged 40–49 years. The participation rate for women aged 30–59 years was 52.1%. Women who were married, had 6–12 years of schooling, lived in urban areas, were unemployed, had insurance coverage, and were in lowest socio-economic quintile, showed greater participation in cervical cancer screening program. All socio-demographic variables were significantly associated with cervical cancer screening in the bivariate analysis, except for the age (Table 1).

Table 2 shows participation rates in each province as a whole, and by urban and rural populations. As shown, highest rates were obtained in the provinces of Isfahan, Fars, and Kohgiluyeh and Boyer-Ahmad. In contrast, Sistan and Baluchestan, Hormozgan, and Kerman had lowest participation rates. Urban-rural disparity in participation rate was greatest in Khorasan-Razavi and Ilam, while being smallest for Fars and Kohgiluyeh and Boyer-Ahmad.

**Table 2:** Cervical Cancer Screening Participation of Women aged 18 and above by Province and Urban-Rural Areas

Province	n	Provincial Participation rate	Urban Participation rate	Rural Participation rate	***P***-value
**Alborz**	511	44.4%	45.8%	31.3%	0.054
**Ardabil**	231	36.8%	40.0%	30.9%	0.169
**Bushehr**	209	37.8%	42.3%	29.2%	0.062
**Chaharmahaal and Bakhtiari**	330	42.7%	38.8%	48.5%	0.079
**East-Azerbaijan**	677	34.7%	36.6%	30.7%	0.134
**Fars**	938	54.8%	55.0%	54.5%	0.884
**Gilan**	485	43.9%	46.6%	39.6%	0.127
**Golestan**	304	29.3%	32.1%	26.2%	0.261
**Hamadan**	344	47.1%	52.4%	39.1%	0.015
**Hormozgan**	304	25.3%	32.3%	18.1%	0.005
**Ilam**	231	45.9%	53.6%	34.4%	0.004
**Isfahan**	985	61.2%	61.1%	62.2%	0.798
**Kerman**	535	24.5%	32.1%	14.1%	< 0.001
**Kermanshah**	384	44.3%	45.6%	41.5%	0.447
**Khorasan-Razavi**	1097	37.8%	42.6%	25.6%	< 0.001
**Khuzestan**	799	34.4%	36.3%	30.2%	0.095
**Kohgiluyeh and Boyer-Ahmad**	275	53.5%	53.8%	53.0%	0.892
**Kurdistan**	298	37.2%	41.6%	28.1%	0.025
**Lorestan**	332	35.2%	37.9%	31.0%	0.198
**Markazi**	276	42.8%	46.2%	33.8%	0.060
**Mazandaran**	581	44.4%	46.8%	41.7%	0.219
**Qazvin**	231	52.4%	50.0%	58.0%	0.267
**Semnan**	247	40.1%	39.2%	43.1%	0.591
**Sistan and Baluchestan**	409	7.6%	6.8%	8.3%	0.561
**South-Khorasan**	618	35.4%	39.4%	31.2%	0.033
**Tehran**	2313	51.4%	51.5%	49.7%	0.634
**West-Azerbaijan**	578	25.4%	28.0%	20.8%	0.055
**Yazd**	358	41.3%	41.1%	42.6%	0.823
**Zanjan**	380	32.6%	36.1%	26.8%	0.059
**Total**	15,260	41.6%	44.7%	34.3%	< 0.001

*N* number of women interviewed in each province; *P*-value obtained from Pearson Chi-Square test

The results of the multivariate analysis of the factors associated with participation in cervical cancer screening are presented in Table 3. Women who were married, had 6–12 years of schooling, and were covered by a health insurance plan were most likely to participate in the cervical cancer screening program. Employment was a negative predictor for service utilization, and age was insignificantly associated with participation. Uptake rates were higher in lower socio-economic levels. After accounting for other covariates in the model, residing area was no more a significant predictor for cervical cancer screening participation.

**Table 3:** Logistic Regression Model of Independent Variables Associated with Cervical Cancer Screening Participation in Iranian Women

Variable	aOR	95% Confidence Interval		***p***-value
		Lower	Upper	
**Age group**				
30–39	1			
40–49	1.11	1.001	1.248	0.48
50–59	0.91	0.803	1.035	0.15
**Marital status**				
Single	1			
Married	45.84	29.460	71.342	< 0.001
Widowed	30.30	18.723	49.056	< 0.001
Divorced	32.82	19.919	54.103	< 0.001
**Education**				
Illiterate	1			
1–6 years	1.76	1.531	2.029	< 0.001
6–12 years	2.47	2.088	2.932	< 0.001
> 12 years	2.24	1.803	2.786	< 0.001
**Employment**				
Unemployed	1			
Employed	.83	.714	.986	0.03
Retired	1.07	.713	1.622	0.72
Student	.92	.423	2.019	0.84
**Primary Insurance**				
No	1			
Yes	1.50	1.245	1.808	< 0.001
**Complementary Insurance**				
No	1			
Yes	1.21	1.072	1.369	< 0.001
**Residence**				
Urban	1			
Rural	1.08	.969	1.216	0.15
**Socio-economic Quintiles**				
Lowest	1			
Low	0.76	0.668	0.883	< 0.001
Middle	0.72	0.630	0.842	< 0.001
High	0.51	0.444	0.604	< 0.001
Highest	0.44	0.377	0.528	< 0.001
**Constant**	0.01			< 0.001

*aOR* adjusted Odds Ratio

## Discussion

This study showed insufficient cervical cancer screening utilization in Iran. About half of the recommended target population in the national guideline (52.1%) have reported having had at least one Pap test in their lifetime. Having 6–12 years of schooling, and being covered under primary or complementary health insurance plans were significantly associated with higher participation. The highest screening uptake rate was obtained in Isfahan and the lowest in Sistan and Baluchestan. Moreover, this study has added to the small body of literature which has assessed socio-economic equity in cervical cancer screening in Iran. A pro-poor bias was observed across the country in cervical cancer screening participation.

In general, the cervical cancer screening participation rate in Iran was low in comparison with most developed countries. In the United States of America 85.5% of women aged 21–64, in Spain 72% of women aged 25–65, and in Australia 61% of women aged 20–69 had undergone cervical cancer screening in the past 3 years [19–21]. However, within developing countries, Iran holds an intermediate position. The prevalence of having ever been screened for cervical cancer was 19% in Jordan (in women aged 20–49), 46.3% in Thailand (in women 30 years and above), and 87.1% in Brazil (in women aged 25–64) [22–25]. Lack of an organized population-based screening program may underlie the lower participation rate in Iran. The opportunistic approach which has been in place for more than three decades, has failed to reach many at-risk women in the population.

The proportion screened was slightly higher for the 40–49-year age group. Nonetheless, age was insignificantly associated with cervical cancer screening participation in the logistic regression analysis. Although this finding was aligned with results published from England and Thailand, but it was inconsistent with those reported in Jordan, and Brazil [22–24, 26]. Probably Iranian women in their thirties, forties, and fifties tend to be equally informed about cervical cancer, and there are no differences in their Pap screening behavior.

As expected, married women were most likely to have undergone cervical screening, which was in line with previous evidence [19, 20, 23]. Married women tend to attend healthcare facilities more often for maternal and child health care which can expose them to awareness activities about cervical cancer screening. Yet, there are several possible explanations for the very low participation rate in single women shown in this study. Sexual relationship outside marriage is not culturally accepted in Iran. Therefore, single women are commonly less sexually active. And, those who are sexually active may not accept to be screened with a test which is perceived to be for married women, in fear of the potential social stigma. Moreover, the participation rate in single women may also be more affected by under-reporting due to embarrassment in admitting to have been screened for cervical cancer.

With regards to education level, women with no education were least likely to participate in cervical cancer screening program. Higher education was associated with higher participation only until about 12 years of schooling. This finding is consistent with other study results conducted in Iran, but not with those of other countries [14, 19, 20]. The fact that illiteracy was associated with lower level of participation, reflects the existing problems in effective communication. Problems which result in difficulties in understanding the benefits of screening in women with no education.

The present study determined that employment was a negative predictor for cervical cancer screening practice, when compared to unemployment. While, participation rates in retired women and university students did not differ significantly from that of unemployed women. This finding contradicts similar studies in other countries, yet validates the results of studies conducted in Iran [14, 27, 28]. Having less free time, might be the possible rationalization for this unexpected finding among working women. Furthermore, hours of operation in most healthcare facilities delivering cervical screening services coincide with usual working hours and may act as a barrier for employed women.

In parallel with other studies, women with health insurance coverage were more likely to have undergone cervical screening [20, 29, 30]. Health insurance plans can increase health service utilization by reducing out-of-pocket expenditures and alleviating potential financial barrier to the service uptake. In Iran, the basic health insurance plans cover most cervical cytology screening costs, and complementary plans provide a full coverage of the service fees.

Uptake rates in urban and rural settlements showed no statistically significant difference in the multivariate analysis. Controversial results have been reported in the literature. In Thailand, rural populations have reported greater participation in cervical cancer screening program. Whereas in Spain and Jordan, the prevalence of cervical cancer screening was higher in urban settings. In Canada, participation rates were not significantly different for urban and rural residents [20, 22, 23, 31]. This finding points to the considerable success of the Iranian primary health care approach in delivering health services to remote and rural areas of the country in the past three decades [32].

This study also revealed evidence of disparities in cervical cancer screening participation across socio-economic groups in Iran. Contradicting with findings in most countries, women with higher socio-economic status had shown lower participation in cervical cancer screening program [19, 22]. However, pro-poor inequality was shown in out-patient healthcare utilization in multiple studies in Iran [33, 34]. Perhaps this finding could be explained by the nature of the health care system. In Iran, the primary health care facilities are all publicly owned. Women in lower socio-economic levels routinely visit these facilities for healthcare services such as maternal and child care, which provides opportunities for communication about cervical cancer screening. Whereas, women in higher socio-economic levels often see specialists directly and based on their health complaints. Therefore, preventive services are substantially underutilized by this group [35].

The major strength of the study is the use of a large nationally representative sample of rural and urban Iranian women, containing different socio-economic levels, that allowed more confident inferences about the population. However, there are some potential limitations as well. First, the cross-sectional design of the study has limited the ability to draw conclusions about causal relationships. Second, the data on the history of having ever undergone cervical cytology were self-reported, and therefore may be susceptible to recall and social desirability biases. Lastly, by evaluating secondary data sources in this study, the assessment of all factors associated with cervical cancer screening participation was not possible.

## Conclusion

Considering the findings of this study, it can be concluded that participation rate for cervical cancer screening is not optimal in Iran and could be improved. Moreover, with regard to the distribution of cervical cancer screening practice, social and geographical disparities have been determined which indicate the need for further research and more comprehensive strategies in order to reduce them.

## Data Availability

The data that support the findings of this study are available from National Institute of Health Research (NIHR) but restrictions apply to the availability of these data, which were used under license for the current study, and so are not publicly available. Data are however available from the authors upon reasonable request and with permission of National Institute of Health Research (NIHR).
